# Individualized cortical gradient and network topology reveal symptom-linked disruptions and neurobiological subtypes in schizophrenia

**DOI:** 10.64898/2026.04.25.26351736

**Published:** 2026-04-27

**Authors:** Bin Wan, Sara Larivière, Clara A. Moreau, Varun Warrier, Richard A.I. Bethlehem, Yun-Shuang Fan, Yuankai He, Ingrid Agartz, Stener Nerland, Erik G. Jönsson, Derin Cobia, Lei Wang, Benedicto Crespo Facorro, Rafael Romero-Garcia, Patricia Segura, Nerisa Banaj, Daniela Vecchio, Tamsyn Van Rheenen, Philip James Sumner, Elysha Ringin, Susan Rossell, Sean Carruthers, Philip J. Sumner, Will Woods, Matthew Hughes, Gary Donohoe, Emma Corley, Ulrich Schall, Frans Henskens, Rodney Scott, Patricia Michie, Carmel Loughland, Paul Rasser, Murray Cairns, Bryan Mowry, Stanley Catts, Christos Pantelis, Aristotle Voineskos, Erin Dickie, Henk Temmingh, Freda Scheffler, Oliver Gruber, Rosanne Picotin, Vince D. Calhoun, Kyle M. Jensen, Filip Španiel, David Tomecek, Raymond Salvador, Andriana Karuk, Raymond Salvador, Edith Pomarol-Clotet, Tilo Kircher, Lea Teutenberg, Frederike Stein, Udo Dannlowski, Dominik Grotegerd, Tiana Borgers, Tim Hahn, Rebekka Lencer, Carlos López-Jaramillo, Melissa Green, Yann Quide, Vaughan Carr, Stefan Ehrlich, Peter Kochunov, Christian Sorg, Melissa Thalhammer, David Glahn, Amanda Rodrigue, Kang Sim, Ali Saffet Gonul, Aslihan Uyar Demir, Nicolas Crossley, Alfonso Gonzalez-Valderrama, Philipp Homan, Wolfgang Omlor, Giacomo Cecere, Felice Iasevoli, Giuseppe Pontillo, Raquel Gur, Ruben C. Gur, Kosha Ruparel, Theodore D. Satterthwaite, Scott Sponheim, Caroline Demro, Young Chul Chung, Soyolsaikhan Odkhuu, Albert Yang, I-Jou Chi, Ole Andreassen, Lars T. Westlye, Unn K. H. Haukvik, Nadine Parker, Jakub Kopal, Dag Alnaes, Jaroslav Rokicki, Carl M Sellgren, Maria Lee, Stefan Borgwardt, Mihai Avram, Taeyoung Lee, Hang Joon Jo, Irina Lebedeva, Alexander Tomyshev, Stefan Kaiser, Paul M. Thompson, Theo G. M. van Erp, Jessica A. Turner, Boris C. Bernhardt, Sofie L. Valk, Matthias Kirschner

**Affiliations:** 1. Department of Psychiatry, University Hospitals of Genève, Thonex, Switzerland.; 2. Synapsy Center for Neuroscience and Mental Health Research, University of Genève, Genève, Switzerland.; 3. Max Planck Institute for Human Cognitive and Brain Sciences, Leipzig, Germany.; 4. Institute of Neuroscience and Medicine (INM-7: Brain and Behavior), Research Center Jülich, Jülich, Germany.; 5. Department of Nuclear Medicine and Radiobiology, Universite de Sherbrooke, Montreal, Canada.; 6. Sainte Justine Hospital Azrieli Research Center, Department of Psychiatry and Addictology, University of Montréal, Montréal, QC, Canada.; 7. Department of Psychiatry, University of Cambridge, Cambridge, UK.; 8. Department of Psychology, University of Cambridge, Cambridge, UK.; 9. The Clinical Hospital of Chengdu Brain Science Institute, School of Life Science and Technology, University of Electronic Science and Technology of China, Chengdu, China.; 10. Division of Mental Health and Addiction, Institute of Clinical Medicine, University of Oslo, Oslo, Norway.; 11. Division of Mental Health and Substance Abuse, Diakonhjemmet Hospital, Oslo, Norway.; 12. Centre for Psychiatric Research, Department of Clinical Neuroscience, Karolinska Institutet & Stockholm Health Care Sciences, Stockholm Region, Stockholm, Sweden.; 13. Rotman Research Institute, Baycrest Academy for Research and Education, Toronto, Canada.; 14. Instituto de Biomedicina de Sevilla (IBiS) HUVR/CSIC, CIBERSAM, University of Seville, Seville, Spain.; 15. Laboratory of Neuropsychiatry, Department of Clinical and Behavioral Neurology, IRCCS Santa Lucia Foundation, Rome, Italy.; 16. Department of Psychiatry, University of Melbourne, Melbourne, Australia.; 17. Centre for Mental Health and Brain Sciences, Swinburne University of Technology, Hawthorn, Australia.; 18. School of Psychology, Centre for Neuroimaging, Cognition and Genomics (NICOG), & Galway Neuroscience Centre, University of Galway, Galway, Ireland.; 19. Hunter Medical Research Institute, Newcastle, Australia.; 20. Faculty of Health, Medicine and Behavioural Sciences, University of Queensland, Brisbane, Australia.; 21. Department of Psychiatry, University of Melbourne, Carlton South, VIC, Australia.; 22. Campbell Family Mental Health Research Institute, CAMH, Toronto, Canada.; 23. Department of Psychiatry, Temerty Faculty of Medicine, University of Toronto, Toronto, Canada.; 24. Neuroscience Institute, University of Cape Town, Cape Town, South Africa.; 25. Neuroscience Institute, University of Cape Town, Cape Town, South Africa.; 26. Section for Experimental Psychopathology and Neuroimaging, Department of General Psychiatry, Heidelberg University, Heidelberg, Baden-Württemberg, Germany.; 27. The Tri-Institutional Center for Translational Research in Neuroimaging and Data Science (TReNDS), Georgia State University, Georgia Institute of Technology, Emory University, Atlanta, GA, USA.; 28. National Institute of Mental Health, Klecany, Czech Republic.; 29. FIDMAG Germanes Hospitalàries Research Foundation, Barcelona, Spain.; 30. CIBERSAM, ISCIII, Barcelona, Spain.; 31. Department of Psychiatry, Marburg University, Marburg, Germany.; 32. Institute for Translational Psychiatry, University of Münster, Münster, Germany.; 33. Department of Psychiatry, Medical School and University Medical Center OWL, Protestant Hospital of the Bethel Foundation, Bielefeld University, Bielefeld, Germany.; 34. German Center for Mental Health (DZPG), Site Jena Magdeburg Halle, Germany.; 35. Center for Intervention and Research on Adaptive and Maladaptive Brain Circuits Underlying Mental Health (C-I-R-C), Site Jena Magdeburg Halle, Germany.; 36. Research Group in Psychiatry, Department of Psychiatry, School of Medicine, Universidad de Antioquia, Medellin, Colombia.; 37. School of Clinical Medicine, Discipline of Psychiatry and Mental Health, UNSW Sydney, Sydney, NSW, Australia.; 38. Translational Developmental Neuroscience Section, Division of Psychological and Social Medicine and Developmental Neurosciences, Faculty of Medicine, TU Dresden, Deresden, Germany.; 39. Maryland Psychiatric Research Center, Department of Psychiatry, University of Maryland School of Medicine, Baltimore, MD, USA.; 40. Department of Diagnostic and Interventional Neuroradiology, School of Medicine and Health, TUM Klinikum Rechts der Isar, Technical Universtiy of Munich, Munich, Germany.; 41. Department of Psychiatry, School of Medicine and Health, TUM Klinikum Rechts der Isar, Technical Universtiy of Munich, Munich, Germany.; 42. Department of Psychiatry, Boston Children’s Hospital, Harvard Medical School, Harvard University, Boston, MA, USA.; 43. West Region, Institute of Mental Health, Singapore, Singapore.; 44. Ege University Hospital, Psychiatry Department, Izmir, Turkiye.; 45. Department of Psychiatry, Pontificia Universidad Católica de Chile, Chile.; 46. School of Medicine, Finis Terrae University, Chile; and Early Intervention Service (PROITP), Dr José Horwitz Barak Psychiatric Institute, Santiago, Chile.; 47. Department of Psychiatry, Psychotherapy and Psychosomatics, Psychiatric Hospital University of Zurich, Zurich, Switzerland.; 48. Department of Neuroscience, University of Naples “Federico II”, Naples, Italy.; 49. Department of Advanced Biomedical Sciences, University of Naples “Federico II”, Naples, Italy.; 50. Perelman School of Medicine, University of Pennsylvania, Philadelphia, PA, USA.; 51. Department of Psychiatry and Behavioral Sciences, University of Minnesota, Minneapolis, MN, USA.; 52. Department of Psychiatry, Jeonbuk National University, Medical School, Jeonju, Korea.; 53. Digital Medicine and Smart Healthcare Research Center, National Yang Ming Chiao Tung University, Taipei, Taiwan.; 54. Institute of Brain Science, National Yang Ming Chiao Tung University, Taipei, Taiwan.; 55. Department of Psychiatry, Taipei Veterans General Hospital, Taipei, Taiwan.; 56. Department of Medical Research, Taipei Veterans General Hospital, Taipei, Taiwan.; 57. School of Medicine, National Yang Ming Chiao Tung University, Taipei, Taiwan.; 58. Department of Occupational Therapy, Kaohsiung Medical University, Kaohsiung City, Taiwan.; 59. Biomedical Artificial Intelligence Academy, Kaohsiung Medical University, Kaohsiung City, Taiwan.; 60. Centre for Precision Psychiatry, Division of Mental Health and Addiction, Oslo University Hospital & University of Oslo, Oslo, Norway.; 61. KG Jebsen Centre for Neurodevelopmental Disorders, University of Oslo, Oslo, Norway.; 62. Department of Psychology, University of Oslo, Oslo, Norway.; 63. Adult Psychiatry Department, Division of Mental Health and Addiction, Institute of Clinical Medicine, University of Oslo, Oslo, Norway.; 64. Centre for Research and Education in Forensic Psychiatry (SIFER), Oslo University Hospital, Oslo, Norway.; 65. Department of Electronic Systems, Vilnius Tech, Vilnius, Lithuania.; 66. Department of Physiology and Pharmacology, Karolinska Institutet, Stockholm, Sweden.; 67. Centre for Psychiatric Research, Department of Clinical Neuroscience, Karolinska Institutet & Stockholm Health Care Sciences, Stockholm Region, Stockholm, Sweden.; 68. Department of Psychiatry and Psychotherapy, Center of Brain, Behavior and Metabolism (CBBM), University of Lübeck, Lübeck, Germany.; 69. Department of Psychiatry, Kyungpook National University, Daegu, South Korea.; 70. Department of Physiology, College of Medicine, Hanyang University, Seoul, South Korea.; 71. Mental Health Research Center, Moscow, Russian Federation.; 72. Imaging Genetics Center, Stevens Institute for Neuroimaging & Informatics, Keck School of Medicine, University of Southern California, Los Angeles, CA, USA.; 73. Department of Psychiatry and Human Behavior, University of California Irvine, Irvine, CA, USA.; 74. Department of Psychiatry and Behavioral Health, the Ohio State University, Columbus, OH, USA.; 75. McConnell Brain Imaging Centre, Montréal Neurological Institute-Hospital, McGill University, Montréal, QC, Canada.; 76. Institute of Systems Neuroscience, Heinrich Heine University Düsseldorf, Düsseldorf, Germany.

**Keywords:** Schizophrenia, Cortical thickness, Structural covariance network, Cortical gradients, Small-world topology, Subtype

## Abstract

Schizophrenia is often conceptualized as a brain network disorder, yet the organizational principles and heterogeneity underlying widespread cortical abnormalities remain poorly understood. Leveraging multisite MRI data from 3,958 individuals diagnosed with schizophrenia and 5,489 neurotypical individuals, we studied the cortical organization and its subtyping by analyzing individualized cortical network similarity. We used eigenvector decompositions to study spatial patterning of the gradients and graph theory to study small-world topology. Individuals with schizophrenia showed widespread alterations of gradient loadings, which followed inferior–superior and frontal–temporal axes. Alterations in small-world topology were localized in key network hubs, including the insula and anterior cingulate cortex. Brain-symptom association analyses identified a latent dimension linking disorganization symptoms to topological alterations. Finally, clustering cortical alterations identified two robust subtypes, characterized by divergent anterior cingulate (S1) versus temporoparietal (S2) thickness differences aligned with the intrinsic gradient-topology patterns. Both subtypes were present early in the illness and stable across disease stages and age groups. These findings reveal systematic disruptions of cortical organization in schizophrenia, providing a network-level framework for macroscale brain organization and inter-individual heterogeneity.

## Introduction

Schizophrenia is a severe mental disorder characterized by heterogeneous symptom dimensions, including positive, negative, affective, and cognitive symptoms, and highly variable illness trajectories (Jeste, Wolkowitz, and Palmer 2011; Levine et al. 2011; Millan et al. 2016; Starzer et al. 2023; Van Rheenen et al. 2026; Velthorst et al. 2016). Neuroimaging studies consistently demonstrate widespread cortical thickness alterations (Theo G. M. van Erp et al. 2018; Georgiadis et al. 2024; Schultz et al. 2010; Wannan et al. 2019; Zhao et al. 2022) with variations related to symptom profile and illness stage (Georgiadis et al. 2024; Jiang, Luo, et al. 2024; Kirschner et al. 2020; Nerland et al. 2025). This heterogeneity extends beyond clinical presentation to the brain itself: patterns of cortical alteration vary markedly across individuals with schizophrenia (Alnæs et al. 2019; Brugger and Howes 2017; Chand et al. 2020; Heilbronner et al. 2016; Omlor et al. 2025; Savaré et al. 2023; Segal et al. 2023; Velthorst et al. 2016; Zhang et al. 2015). Recent work has begun to capture this neuroanatomical diversity by identifying two subtypes of schizophrenia, characterized by predominant frontal versus temporal–hippocampal alterations, with morphometric differences in both subtypes subsequently extending across the cortex (Jiang, Luo, et al. 2024). Moreover, gray-matter heterogeneity appears most pronounced near illness onset and diminishes with chronicity (Jiang et al. 2025).

Advances in network neuroscience have demonstrated that cortical changes are not isolated phenomena but are embedded within large-scale patterns of inter-regional covariance (Alexander-Bloch et al. 2013; Ed Bullmore and Sporns 2009; Sebenius et al. 2025; Seidlitz et al. 2018; Wannan et al. 2019; Yun et al. 2016, 2020). The spatial organization and topology of these covariance patterns can be interrogated with two complementary frameworks. First, eigenvector decomposition approaches capture continuous gradients along which cortical regions are organized according to shared covariance profiles, reflecting fundamental principles of the cortical organization (Margulies et al. 2016; Vos de Wael et al. 2020). In healthy young adults, dominant gradients of cortical thickness covariance align with anterior–posterior and inferior–superior axes of cortical organization (Valk et al. 2020). Second, graph-theoretic analyses of structural covariance characterize network topology, including small-world organization, which captures the balance between local specialization and global integration (Ed Bullmore and Sporns 2009; Humphries and Gurney 2008; Watts and Strogatz 1998). Deviations from this balance are interpreted as network disruptions and have been linked to a variety of psychiatric and neurological disorders (Larivière et al. 2022; Sha et al. 2022; Yun et al. 2020).

Through neurodevelopment, coordinated cortical changes follow systematic trajectories informed by evolutionary principles (Shaw et al. 2008). For example, tangential versus radial patterns of progenitor cell division in the embryonic cortex (Fernández, Llinares-Benadero, and Borrell 2016) implies large-scale anterior-posterior and inferior-superior gradients of cortical thickness across species (Valk et al. 2020). Beyond early development, cortical structure continues to change across the lifespan (Bethlehem et al. 2022) and is shaped by shared genetic influences across distributed regions, resulting in coordinated variation in cortical features such as thickness and area (Elliott et al. 2018; Grasby et al. 2020). Together, these findings suggest that deviations in local cortical properties are unlikely to occur in isolation, but instead reflect structured, network-level alterations constrained by developmental/genetic architecture. To elucidate how these organizational principles are perturbed in schizophrenia, it is therefore critical to characterize cortical organization by studying macroscale gradients and network topology (Fan et al. 2025; Georgiadis et al. 2024; Kirschner et al. 2020; Shafiei et al. 2020; Shao et al. 2024).

Building on these developmental and organizational principles, cortical covariation networks are increasingly studied in neurodevelopmental and psychiatric disorders, including schizophrenia (Edward Bullmore 2019; Fan et al. 2025; Prasad et al. 2022; Shao et al. 2024; Yun et al. 2016). For example, a compressed anterior-posterior cortical gradient has been reported in early schizophrenia at the group level (Fan et al., 2025), while network topology analyses have revealed alterations in clustering within key hub regions such as the insula in adult schizophrenia (Shao et al. 2024). Furthermore, emerging inter-individual covariance approaches (Sha et al. 2022; Yun et al. 2020) show that deviations in cortical organization relate to clinical symptoms, highlighting their potential relevance for understanding disease heterogeneity (Liu et al. 2021). Together, these findings motivate an integrative framework that combines macroscale cortical gradients and network topology to characterize cortical covariation architecture and inter-individual variability in schizophrenia.

**Fig. 1:**
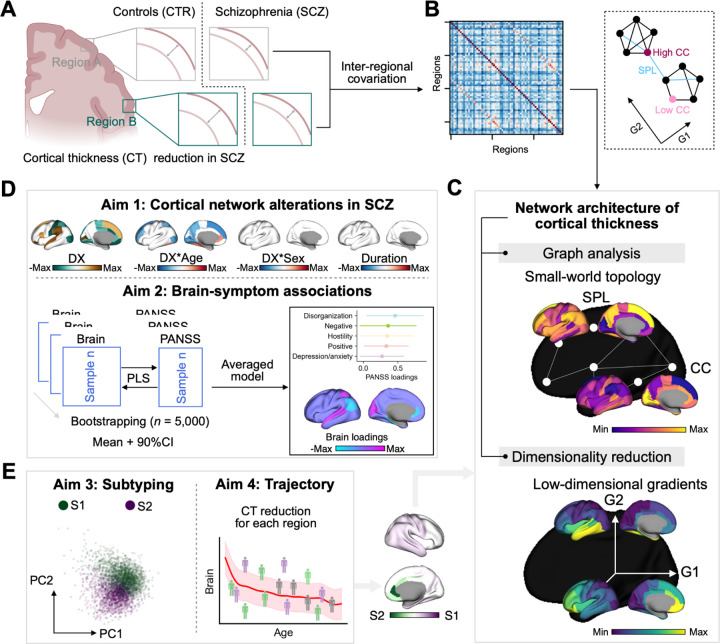
Conceptualization and aims of the study. **A:** Individuals with schizophrenia experienced extensive cortical thickness reduction. **B:** Group-level inter-regional covariance matrix is calculated by inter-subject Pearson correlation, which can be used to describe the structural network systems of cortical thickness. We can understand the matrix by reducing the dimensionality or studying the graph topology. G1 and G2 are two principal components. SPL indicates *shortest path length* between two regions and CC indicates *clustering coefficient* of a region, i.e., how many edges exist for a region. **C**: Brain maps for group-level low-dimensional gradients and small-world topology in healthy controls. All analyses were performed using the top 20% values of covariance matrix per row. **D**: In order to compare between individuals with schizophrenia and controls, we used the individual covariance formula as suggested by previous studies (Sha et al. 2022; Yun et al. 2020). Aim 1, focus on comprehensive characterization of cortical network alterations in schizophrenia including interaction between diagnosis (DX) and age, interaction between diagnosis (DX) and sex, and disease duration related brain maps. In aim 2, we examine the latent brain-symptom associations between cortical network architecture features and multiple clinical symptom dimensions, measured with the Positive and Negative Syndrome Scale for Schizophrenia (PANSS) using multivariate partial least square (PLS) approach with 5,000 bootstrap resamples. **E**: To address neuroanatomical heterogeneity in schizophrenia, we apply data-driven clustering to the individual-level gradient-topology profiles to uncover reproducible schizophrenia subtypes (Aim 3). We then use deviation from population modeling, to identify subtype-specific cortical-thickness profiles across the adult lifespan (Aim 4).

Here, we analyzed multisite brain MRI data from 9,447 individuals across 40 international sites from the ENIGMA Schizophrenia Working Group to comprehensively map individual cortical network organization in schizophrenia. We constructed group- and individual-level cortical thickness covariance and distance networks and derived macroscale cortical gradients together with small-world topological measures (Fig. 1A–C). First, we examined case vs. control differences across gradient and small-world topology to identify systematic disruptions of cortical organization (Fig. 1D). Second, we used multivariate association analyses to link cortical gradient and small-world topology to a shared, dimensional variation in clinical symptoms (Fig. 1D). Third, we applied a clustering approach based on alterations of gradients and small-world topology to identify reproducible cortical subtypes of schizophrenia (Fig. 1E). Finally, we characterized subtype-specific cortical thickness profiles across the adult lifespan and their alignment with macroscale cortical gradient-topology patterns (Fig. 1E).

## Results

### Data samples

We studied 3,958 individuals with schizophrenia (SCZ; 1,488 females, age mean ± SD = 35.0 ± 12.2 years) and 5,489 healthy controls (CTR; 2,783 females, age mean ± SD = 34.6 ± 13.40 years) from 40 centers of the international ENIGMA consortium Schizophrenia Working Group. Diagnosis of schizophrenia or schizoaffective disorder was confirmed at each center using criteria from either the International Classification of Diseases (ICD) or Diagnostic and Statistical Manual (DSM). Details on site-specific demographic and imaging information are provided in **Source Data**.

### Schizophrenia-related alterations in cortical thickness network architecture

**Fig. 2:**
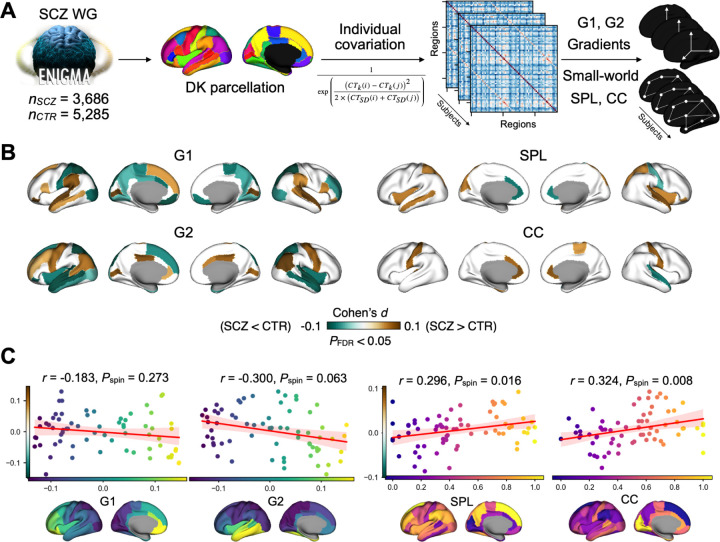
Schizophrenia-related alterations in cortical thickness network architecture. **A**: We extracted FreeSurfer outputs and demographics from the ENIGMA-Schizophrenia (SCZ) consortium including 40 data sites. Cortical thickness (CT) data were parcellated in 68 regions using Desikan-Killiany (DK) atlas (Desikan et al. 2006). The individual variation formula was used to construct the matrix and the individual gradients (G1 and G2) and small-world topology (SPL and CC) were calculated. **B**: Cohen’s *d* brain maps were shown with the threshold of *P*_FDR_ < 0.05 from case-control comparisons. **C**: Spatial correlations between case-control Cohen’s *d* in Fig. 2B and the group-level template maps in Fig. 2C.

We characterized cortical network architecture using principal gradients of dimensionality reduction and small-world topology derived from cortical thickness similarity matrices. We first performed group-level analysis of the covariance matrix to obtain the first two gradients (G1 and G2) and small-world topology (SPL and CC) using sparsity scores ranging from 0.6 (top 40% remained) to 0.9 (top 10% remained) with a 0.01 interval (**Supplementary Fig. S1**). Two principal gradients describing cortical thickness covariance along anterior-posterior (G1) and posterior-temporal (G2) axes were identified, with their order varying by sparsity level and group **(Supplementary Fig. S2**). The temporal anchor of G2 extended into occipital regions, largely overlapping with the anterior–posterior axis described in normative structural covariance studies (Valk et al., 2020). SPL and CC showed reversed spatial patterns, with higher normalized SPL in frontal regions and higher normalized CC in visual regions (**Supplementary Fig. S3**). For all subsequent analysis, we chose a sparsity of 0.8 as it minimized similarity between G1 and G2 (**Supplementary Fig. S1**), and aligns with thresholds used in previous studies (Hettwer et al. 2022; Larivière et al. 2022; Sha et al. 2022). Individual-level gradients were computed using subject-specific difference matrices and aligned to control group-level templates via Procrustes rotation, while individual topology metrics were rescaled to a 0–1 range (Fig. 2A).

Individuals with schizophrenia exhibited system-wide alterations along cortical network gradients and more localized disruptions in small-world topology (Fig. 2B, all *P*_FDR_ < 0.05). For G1, alterations were observed in superior temporal, postcentral, occipital, and prefrontal regions. The observed gradient alterations did not spatially align with the G1 axis and showed a widespread, though randomly distributed, pattern (Pearson *r* = −0.183, *P*_spin_ = 0.273, Fig. 2C). In contrast, alterations along the second gradient (G2), reflecting the frontal–temporal cortical axis, followed a systematic spatial pattern. Affected regions included the left lateral occipital gyrus, bilateral lingual gyrus, superior temporal sulcus and gyrus, precentral gyrus, insula, medial prefrontal areas, and middle cingulate cortex; this disruption pattern closely mirrored the normative structural covariance organization of G2 but without statistical significance (Pearson *r* = −0.300, *P*_spin_ = 0.063, Fig. 2C). Thus, while G1 alterations were randomly distributed along the anterior-posterior axis, G2 alterations showed a more structured disruption pattern along the posterior–temporal axis. Detailed regional statistics were reported in **Source Data**.

Small-world topology disruptions were more spatially constrained but located in key integration hubs. SPL alterations concentrated in the bilateral anterior cingulate cortex, left insula, and superior parietal cortex, while CC changes affected anterior cingulate and postcentral regions. Both topological measures showed significant spatial correlations with their respective normative network patterns (SPL: Pearson *r* = 0.296, *P*_spin_ = 0.016; CC: Pearson *r* = 0.324, *P*_spin_ = 0.008), indicating systematic disruption of local network efficiency (Fig. 2C). Detailed regional statistics were reported in **Source Data**.

We assessed the robustness and sensitivity of cortical network alterations across analytical parameters, including covariate adjustments, sparsity thresholds, and demographic factors (age, sex and duration of illness). First, gradient and topology alterations remained highly consistent with spatial correlation maps between non-ICV and ICV-controlled Cohen’s *d* maps exceeding *r* > 0.98 (**Supplementary Fig. S4**). We further replicated the diagnostic effects of different sparsity parameters (10%, 30%, and 40% of covariance edges, **Supplementary Fig. S5**), where the overall spatial patterns of Cohen’s *d* were similar. Gradients-related regions remained consistent across thresholds, although additional regions emerged at lower sparsity level (i.e., denser matrices). We also examined age × diagnosis (DX) and sex × DX interactions, as well as effects of disease duration **Supplementary Fig. S6**. Significant age × DX interaction along G1 were observed in bilateral occipital and superior parietal cortices, as well as the left prefrontal and anterior cingulate cortex. For G2, age × DX interactions were observed in bilateral prefrontal, superior parietal, and occipital cortex; no regions survived FDR correction for SPL and CC. For sex × DX interactions, no regions survived FDR correction in G2, SPL, or CC, only one region (left precentral gyrus) was significant for G1. No regions showed significant effects of disease duration.

### Dimensional associations with symptom profiles

**Fig. 3:**
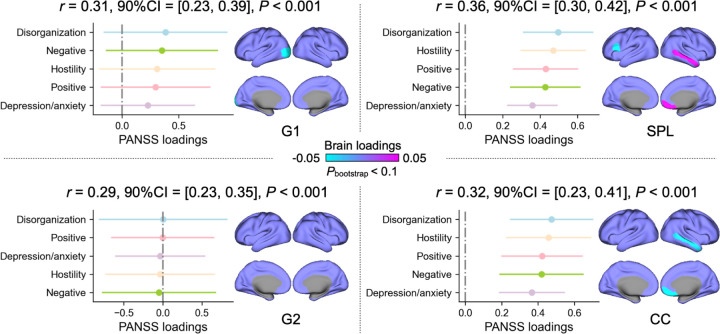
Dimensional associations with clinical profiles. Multivariate brain-symptom associations (PLS: partial least squares regression) for G1, G2, SPL, and CC separately were conducted. We selected the averaged model over 5,000 bootstrap resamples. We used 90% confidence interval (CI) to test the significance of brain and clinical features, which is *P* < 0.1.

To determine how cortical network architecture relates to clinical heterogeneity in schizophrenia, we examined associations with symptom dimensions using multivariate partial least squares (PLS) analysis in the subsample with available item-level PANSS data (*n* = 881, Fig. 3). While gradient measures showed no significant associations with clinical symptoms, both topological measures exhibited robust symptom-brain relationships across all five PANSS factors: positive, negative, cognitive/disorganization, depression/anxiety, and hostility (Lim et al. 2021).

The overall brain-symptom association was robust at the model level (*P*_bootstrp_ < 0.001), though individual feature loadings did not survive at the conventional 95% confidence level and are therefore reported at the 90% confidence interval as exploratory. The strength of these associations followed a clear hierarchy, with depression/anxiety showing the weakest loadings and disorganization the strongest. Regionally, SPL and CC both converged on the right middle temporal cortex and medial orbitofrontal cortex, but each also showed distinct positive loading patterns: SPL was additionally associated with the left inferior frontal cortex, whereas CC did not link to any areas (Fig. 3). Regarding gradients, there was one region, occipital, with significant loadings. These findings suggest that disrupted network efficiency, rather than altered gradients, is directly related to the clinical manifestations of schizophrenia.

The robustness of symptom-topology relationships was further supported by item-level analysis, which preserved both the clinical loading hierarchy and regional specificity (**Supplementary Fig. S7**). Notably, interactions between age or sex with diagnosis were minimal, with only sparse regional effects surviving correction, indicating stability of network-symptom relationships across these demographic variables.

### Two subtypes identified by altered network architecture in schizophrenia

**Fig. 4:**
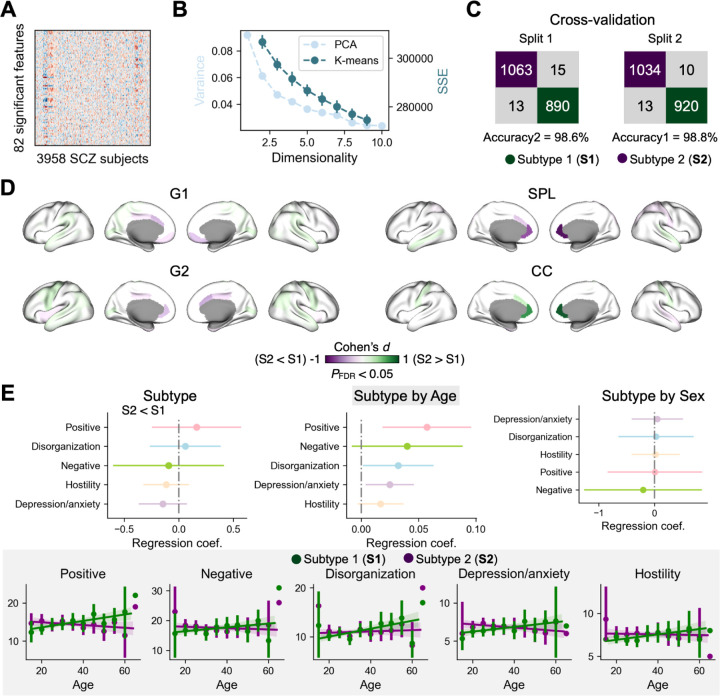
Subtypes in schizophrenia. **A**: The input matrix of 82 significant features from case-control comparisons in Fig. 2B in 3,958 individuals with schizophrenia. **B**: Principal component analysis (PCA) was performed on the matrix to reduce the dimensionality of brain features. The first two PCs explained 9.9% and 9.2% of the total variance. K-means was performed on the matrix to cluster subjects into subtypes. K cluster number ranges from 2 to 9. Sum of squared errors (SSE) indicate the sum of distance between each data point and its assigned centroid. **C**: Cross-validation of the two subtypes (S1 and S2) by 1:1 splitting the whole sample into two subsamples. Both assignment concordances were greater than 98%. **D**: Subtype difference in gradients and small-world topology (82 features from **A**). **E**: Subtype difference in schizophrenia clinical factors as well as interactions of subtype with age and sex. We additionally showed the age correlation with symptom profiles in S1 and S2 separately given the significant subtype-by-age interactions.

Next, to study the heterogeneity of the systematic disruptions of cortical organization in schizophrenia, we clustered these individuals using 82 significant features identified in the case–control analysis from Fig. 2B, which delineates biologically meaningful subtypes in schizophrenia (*n* = 3,958, Fig. 4A).

A *k*-means clustering algorithm was first applied to this individual-by-feature matrix (Fig. 4B), but the sum of squared errors (SSE) curve did not show clear convergence along 2–9 clusters. To improve separability, we reduced feature dimensionality using principal component analysis (PCA, **Supplementary Fig. S8**) to help identify the ideal number of subtypes. Overall, principal components 1–5 accounted for 9.18%, 6.13%, 4.70%, 4.20%, and 3.63% of the total variance. In this space, a two-subtype solution (S1 and S2) showed a clear separation of the data from the scatter plot (**Supplementary Fig. S8**). Robustness of the two subtypes was confirmed by half-split cross-validations, yielding assignment concordances of 98.6% and 98.8% across splits (Fig. 4C). In total, 2,125 individuals (53.7%) were categorized as S1 and 1,833 (46.3%) as S2. There were no significant differences in age (*t* = 1.68, *P* = 0.093), sex ratio (chi-square = 0.66, *P* = 0.415), or disease duration (*t* = 0.62, *P* = 0.532) between subtypes.

We observed significant subtype differences across all 82 brain network features (Fig. 4D) mirroring the case-control difference in gradient and network topology observed when comparing the entire schizophrenia sample with controls (Fig. 2B). Direct comparison between S1 and S2 revealed that S2 exhibited a more schizophrenia-like pattern while the pattern of S1 was relatively similar to the control group (see Fig. 2B). Subtypes did not differ in the five symptom factors. However, in the linear regression of *symptom ~ age + sex + subtype + age × subtype*, there were significant interactions between subtype and age in positive (*t* = 2.95, *P* = 0.003), disorganization (*t* = 2.10, *P* = 0.036), and depression/anxiety symptoms (*t* = 2.40, *P* = 0.016), shown in Fig. 4E. In particular, S1 showed a positive correlation between age and symptoms (positive: Pearson *r* = 0.146, *P* = 0.003; disorganization: Pearson *r* = 0.177, *P* < 0.001; depression/anxiety: Pearson *r* = 0.102, *P* = 0.037), whereas correlations for S2 were negative but did not reach statistical significance.

Item-level analyses revealed that subtypes did not differ on PANSS items (**Supplementary Fig. S9**). Subtype × age interactions were significant in delusions, unusual thought content, anxiety, preoccupation, conceptual disorganization, guilt, active social avoidance, lack of judgement insight, and grandiosity. No significant subtype × sex interactions were observed for any PANSS item.

Together, *k*-means clustering based on cortical network architecture features identified two subtypes that differ robustly in gradient organization and network topology, as well as age correlations with symptom dimensions.

### Cortical thickness reduction differs between two subtypes

**Fig. 5:**
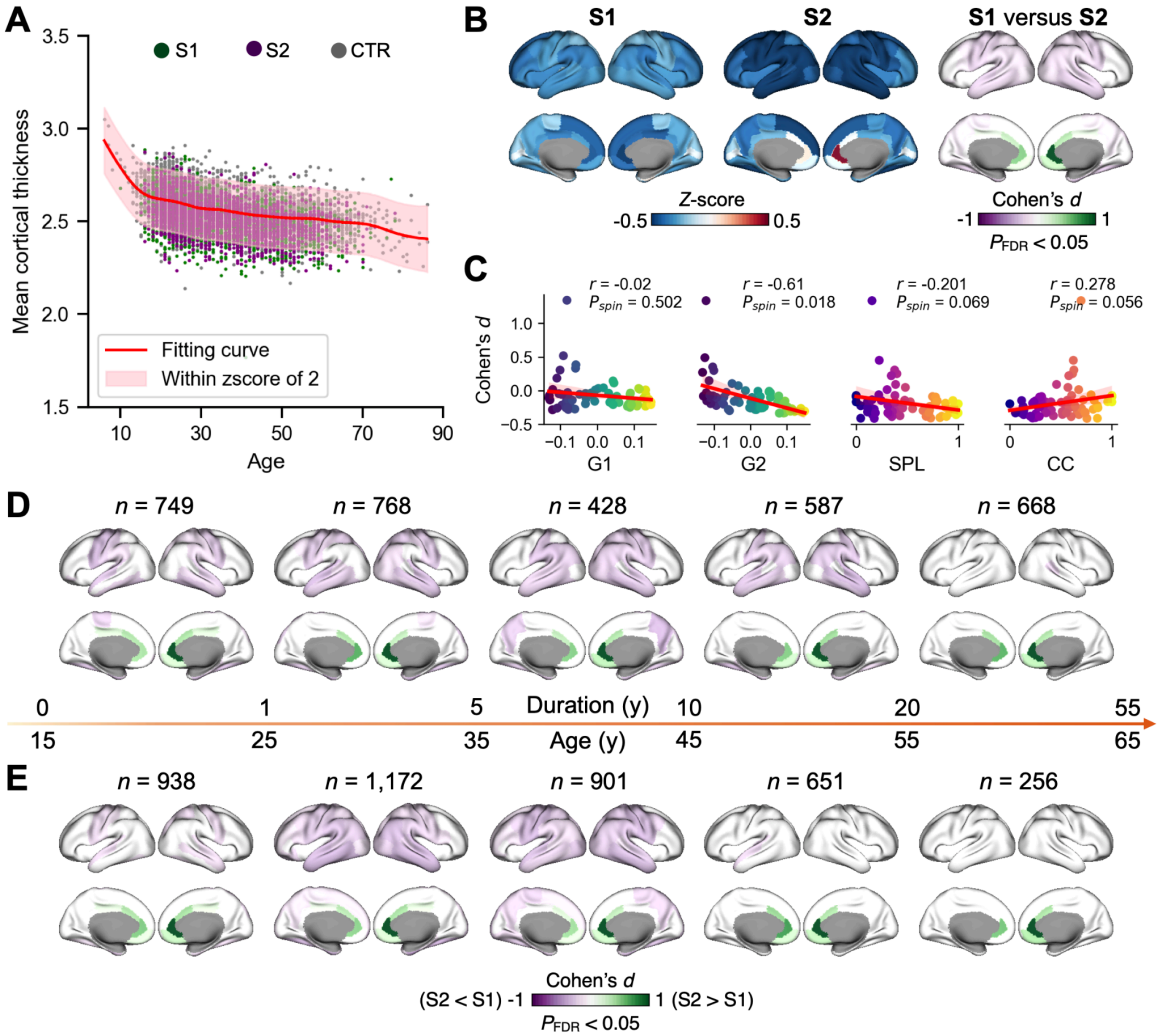
Cortical thickness profiles of subtypes. **A**: Normative model of mean cortical thickness using healthy controls data (*n* = 5,285). Deviation scores (z-scores) were derived from the normative model for each individual including schizophrenia and healthy control. **B**: Using regional normative models, we extracted z-scores for each region and individual. We compared z-scores between subtype 1 (S1) and subtype 2 (S2). **C:** We also correlated the S1 versus S2 z-score difference (Cohen’s *d*) map with the group level gradient-topology patterns from the controls in our cohort. We visualized the difference maps in different disease duration (**D**) and age (**F**) groups. Disease duration was categorized into five groups: 0–1, 1–5, 5–10, 10–20, and 20–55 years. Age was categorized into five groups from 15 to 65 years with a 10-year interval.

Next, we used generalized additive models (GAM) to examine how the cortical thickness profiles of the two subtypes deviate from normative trajectories of cortical thickness across the adult lifespan. We first fitted GAM for mean cortical thickness in controls and extracted individual deviation scores (*z*-scores). Heterogeneity was defined as the proportion of individuals showing extreme deviations (i.e., |z-score| > 2). Compared to controls (*z*-score = 0.00±1.02, heterogeneity = 4.5%), both subtypes showed lower mean cortical thickness z-scores (S1: *z*-score = −0.53±1.05, heterogeneity = 9.3%; S2: *z*-score = −0.66±1.13, heterogeneity = 11.7%). Mean z-scores differed significantly between S1 and S2 (S1 vs S2: *t* = 3.63, *P* = 0.0003) as well as between each subtype and controls (S1 vs controls: *t* = 20.53, *P* = 3.49e-91; S2 vs CTR: *t* = 23.61, *P* = 7.31e-119, upper right corner of Fig. 5A).

In a second step, we applied GAM for each cortical region to identify subtype-specific profiles of regional deviations from the normative cortical thickness patterns. In particular, S1 showed a cortical thinning pattern across the whole cortex, while S2 showed cortical thinning in medium frontal, temporal, parietal, and central cortices but increased thickness in anterior cingulate cortex (Fig. 5B). Comparing regional z-scores between S1 and S2, revealed that S2 showed significantly thinning in the temporal, central, and parietal regions relative to S1, while S1 showed thinning in the anterior cingulate cortex relative to S2 (Fig. 5B, *P*_FDR_ < 0.05). These subtype differences in regional cortical thickness were highly spatially correlated with group level gradient-topology patterns of G2 (G1, Spearman *r* = −0.020, *P*_spin_ = 0.502; G2, Spearman *r* = −0.610, *P*_spin_ = 0.018; SPL, Spearman *r* = −0.201, *P*_spin_ = 0.069; CC, Spearman *r* = 0.278, *P*_spin_ = 0.056; Fig. 5C).

Finally, we examined whether cortical thickness differences between S1 and S2 were evident at early illness stages and whether these subtype-specific profiles relate to disease duration or age. Stratifying patients by duration of illness (0–1, 1–5, 5–10, 10–20, and 20–55 years) showed that regional z-score differences between S1 and S2 were already detectable in the earliest stage (0–1 year) and remained robust across first four subsequent duration bins (FDR-corrected; Fig. 5C, see **Supplementary Fig. S10C** for subtype-specific mean z-score maps for each duration bin). The observed effects in temporal and parietal regions were not significant in the last bin, but the anterior cingulate cortex finding remained. The spatial pattern of subtype differences was qualitatively consistent across stages, indicating that these cortical profiles emerge early in the illness course and persist throughout its progression.

Complementary regression models testing duration by subtype interactions in mean and regional cortical thickness were not significant (*P*_FDR_ > 0.1), suggesting comparable trajectories across subtypes (**Supplementary Fig. S10A-B)**.

A complementary age-stratified analysis (15–65 years, 10-year intervals) revealed a similar pattern: regional z-score differences between S1 and S2 remained significant across the first three age bins (FDR-corrected; Fig. 5D, see **Supplementary Fig. S11C).** Age bins after 45 years were mainly significant in the anterior cingulate cortex. The spatial distribution of subtype differences closely resembled that observed across bins of illness-duration, and no significant interactions between age × subtype or sex × subtype were detected (**Supplementary Fig. S11A-C**). Together, these findings indicate that distinct cortical thickness profiles of S1 and S2 are established early and remain stable across illness duration, adult age range, and sex.

## Discussion

Leveraging the largest multisite individual-level dataset of schizophrenia to date (~9,000 participants from the ENIGMA Schizophrenia Working Group), we examined individual macroscale cortical network organization by integrating low-dimensional gradients with small-world network topology. Across patients, we observed system-wide alterations of the inferior-superior (G1) and the frontal-temporal (G2) axes of network organization. Cortical gradient alterations in G1 showed a largely random and spatially heterogeneous disruption, whereas alterations in G2 showed a somewhat more systematic compression along the occipital and temporal anchor. In contrast to these widespread gradient alterations, small-world topology showed more localized cases–control differences in key integration hubs including the insula and anterior cingulate cortex. Small-world topology also showed selective and robust brain-symptom associations, with lower CC and higher SPL tracking overall symptom severity, particularly disorganization. These findings point to (i) a core, system-wide gradient reorganization characteristic of schizophrenia, and (ii) a topological component mediating individual differences in symptom expression. Finally, the data-driven clustering of the disorder-specific gradient-topology features identified two reproducible biological subtypes reflecting the whole cortex (**S1**) and [temporal, central, medium frontal, and parietal] (**S2**) patterns of reduced cortical thickness. Comparison of cortical profiles further differentiated the subtypes, with anterior cingulate predominance in S1 and posterior predominance in S2. These differential subtype-specific cortical profiles aligned along the group-level gradient-topology patterns, emerged early in the illness course (<1 year of duration), and remained detectable across the full adult age range, independent of sex. Together, these findings delineate systematic cortical network disruptions in schizophrenia, encompassing widespread gradient alterations and topology–symptom associations, alongside organization-derived subtypes with distinct cortical profiles that arise early and remain stable across the adult lifespan.

At the level of cortical organization, schizophrenia was characterized by widespread disruption of structural covariance gradients. The inferior-superior gradient (G1) displayed a largely random pattern of change, whereas the frontal-temporal gradient (G2) showed a compression-type shift in gradient organization. Developmentally, this compression may reflect delayed or incomplete maturation of anterior association cortices (Fan et al. 2025), consistent with neurodevelopmental models of schizophrenia emphasizing disrupted cortical organization formation and impaired prefrontal specialization (Fatemi and Folsom 2009; Tan et al. 2006). Functionally, diminished separation along this axis may underlie the blending of perceptual and higher-order cognitive processes characteristic of the disorder (Uhlhaas and Mishara 2007). The widespread yet spatially heterogeneous disruption along the inferior–superior axis may impair integration between ventral archicortical and dorsal paleocortical developmental streams (Giaccio 2006; Pandya et al. 2015; Valk et al. 2020). Within this dual-origin framework (Valk et al. 2020), such diffuse alterations suggest a failure to maintain the normal differentiation between these cortical lineages, consistent with models of asynchronous maturation and reduced cortical specialization in schizophrenia. From a genetic perspective, these dual cortical origins, as indexed by inverse morphometric inverse divergence, exhibit strong genetic correlation and are linked to schizophrenia risk (Sebenius et al. 2024). This suggests that the observed gradients may be driven, at least largely, by shared genetic architecture.

In contrast to these system-wide gradient alterations, the analysis of small-world topology revealed regionally specific network disruptions centered on key cortical hubs, including the superior parietal, insula, and anterior cingulate cortices. Among these regions, insula is of particular interest, as previous studies have also identified reproducible lower network efficiency (i.e., higher SPL or lower CC) in this region in individuals with schizophrenia (Kim et al. 2020; Shao et al. 2024). Serving as a crucial interface between the temporal and prefrontal regions, both in anatomical terms and in its functional capabilities, insula plays a pivotal role in the discrimination between self-generated and external information (Shao et al. 2024; Wylie and Tregellas 2010). Notably, the broader pattern of topological disruption seen in our study including regions such as the insula, anterior cingulate and superior parietal cortices mirrors prior network-based studies that consistently implicate these regions as central nodes in schizophrenia-related cortical organization (Georgiadis et al. 2024; Jiang, Palaniyappan, et al. 2024; Shafiei et al. 2020). Together, our findings extend prior work by highlighting how localized disruptions of integrative hubs contribute to system-level cortical organization in schizophrenia.

Linking cortical gradients and topology to clinical symptoms revealed a consistent dimensional pattern in which local small-world topology showed the strongest associations with psychopathology. In contrast to the widespread gradient alterations distinguishing patients from controls, symptom severity was preferentially linked to localized topological disruption, suggesting that clinical expression in schizophrenia is driven more by regional network inefficiency than by global shifts in cortical organization. This shared symptom dimension was dominated by disorganization, hostility, positive and negative symptoms with weaker contributions from affective domains. This distinction is clinically relevant, as negative and disorganized symptoms are typically more persistent and prognostically informative than positive symptoms (Fenton and McGlashan 1991; Ho et al. 1998; Mancevski et al. 2007; Starzer et al. 2023), and are associated with poorer functional outcomes and prognosis (Ho et al. 1998; Milev et al. 2005; Rabinowitz et al. 2012; Rocca et al. 2018; Starzer et al. 2023). Rather than mapping onto isolated symptom-specific loci, the identified associations suggest a shared symptom dimension supported by localized topological disruption across a set of key cortical regions, including middle temporal, inferior frontal, and orbitofrontal areas. These regions are implicated across multiple studies of disorganization, positive and negative symptoms and are central to executive control, semantic integration, and self-monitoring processes (Franz et al. 2025; Palaniyappan et al. 2020; Sharkey et al. 2024; Walton et al. 2018). From a network perspective, this pattern is consistent with the idea that local module-level inefficiency or disrupted hub integration in centrally positioned regions may have disproportionate effects on cognitive organization and goal-directed behavior, thereby giving rise to clinically salient symptoms. Thus, while cortical gradients capture broad, system-level reorganization distinguishing patients from controls, network topology appears to provide a more proximal substrate linking brain architecture to symptom severity. By identifying a shared, topology-driven symptom dimension dominated by disorganization symptoms, our findings offer a dimensional, network-based framework for understanding how localized disruptions within the cortical connectome contribute to the clinical heterogeneity of schizophrenia.

Finally, using a network-based clustering framework, we identified two robust schizophrenia subtypes characterised by divergent anterior cingulate–posterior profiles of cortical thickness alteration, embedded within the macroscale gradient-topology organisation of the cortex. Neuroanatomically, S2 was characterised by widespread cortical thinning spanning frontal, temporal, parietal, and central cortices with relative preservation of the ACC, whereas S1 showed a more diffuse whole-cortex thinning pattern with pronounced ACC involvement. Clinically, S1 showed an age-related trajectory of increasing positive and disorganization symptoms, potentially reflecting the persistent ACC network disruption observed in this subtype, whereas S2 did not show this age-related increase.

Data-driven subtyping of schizophrenia has been pursued across multiple methodological approaches (Chand et al. 2020; Geisler et al. 2015; Jiang et al. 2023; Jiang, Luo, et al. 2024), including machine learning applied to regional morphometry and sequential staging models such as SuStain, which have identified frontal- and temporal-hippocampal-anchored patterns of cortical alteration (Jiang et al. 2023; Jiang, Luo, et al. 2024). Our findings extend this literature by showing that subtype heterogeneity is organised along the low-dimensional eigenmodes of cortical organization, with the two subtypes reflecting differential weighting of disruptions along the principal gradient-topology axis rather than spatially arbitrary regional abnormalities. This distinction has methodological implications: staging-based approaches such as SuStain (Jiang, Luo, et al. 2024; Young et al. 2018) infer subtypes by ordering abnormalities into hypothetical disease trajectories, assuming monotonic progression, an assumption that remains contested in schizophrenia given its non-linear and heterogeneous illness course (Levine et al. 2011; Starzer et al. 2023). Our gradient-topology framework identifies subtypes based on spatial structure alone, making it robust to the absence of monotonic staging. Future longitudinal studies could directly test whether disease processes unfold along the same network organisational axes identified here, and whether this predicts convergence with staging-based subtyping approaches. Given that cortical gradients capture neurodevelopmental differentiation into specialised systems (Sydnor et al. 2021, 2023), our findings suggest that schizophrenia subtypes may arise from complementary deviations along this organisational axis, ultimately converging toward a shared system-wide alteration pattern. Together, these results advance a network-level reinterpretation of schizophrenia heterogeneity beyond region-centric frameworks..

## Limitations and future directions

While this large-scale international multisite data provide robust and replicable evidence for systematic disruption of cortical network architecture in schizophrenia, the cross-sectional design limits direct mechanistic inference. In particular, population-based developmental cohorts, combined with detailed genetic and environmental risk phenotyping, are critical for elucidating how deviations in cortical network organization emerge prior to illness onset. Such approaches are well suited to disentangle neurodevelopmental processes shaping large-scale cortical architecture from later disease-related network alterations. Complementary, future work in clinical high-risk and early psychosis cohorts may advance how the observed disruptions in gradient and small-world topology manifest closer to the disease phenotype, enabling investigation of early pathological mechanisms within the illness trajectory. In parallel, large-scale genetic resources, including polygenic risk scores, can be leveraged to characterize the genetic architecture of cortical network organization and its overlap with schizophrenia risk. Together, these complementary longitudinal strategies will help bridge normative development, genetic liability, and disease-related altered cortical network organization, advancing mechanistic understanding of schizophrenia at the network level.

## Conclusions

In summary, this study leveraged large-scale and multisite neuroimaging datasets from the ENIGMA Schizophrenia Working Group to provide robust evidence for systematic disruption of cortical gradient and network topology in schizophrenia. At the level of macroscale organization, schizophrenia was characterized by widespread alterations in cortical thickness covariance gradients, reflecting system-level reorganization of cortical architecture. In parallel, disruptions in the small-world network topology were more spatially localized and preferentially linked to clinical symptom expression, particularly disorganization. By integrating gradients and topology, we further identified two robust cortical subtypes with stable anterior-posterior differentiation, supporting the notion that schizophrenia heterogeneity is embedded within large-scale network organization and potentially shaped by developmental/genetic factors. Together, these findings highlight complementary roles of cortical gradients and network topology in capturing both shared organizational alterations and individual variability in symptom expression, offering a network-level framework for understanding individual macroscale cortical organization and its role in shaping clinical heterogeneity in schizophrenia.

## Methods

### Imaging preprocessing

Following published guidelines for ENIGMA pipelines (T. G. M. van Erp et al. 2016; Theo G. M. van Erp et al. 2018), all 36 participating sites processed 3D T1-weighted structural brain MRI scans using the FreeSurfer toolkit (Fischl 2012; Fischl et al. 2002) and extracted cortical thickness from the standard 68 Desikan-Killiany (DK) regions (Desikan et al. 2006). The number of scanners, vendor, strength, sequence, acquisition parameters, and FreeSurfer versions are provided in **Source Data**. Standard ENIGMA protocols for quality control were conducted at each site prior to subsequent mega-analysis (http://enigma.usc.edu/protocols/imaging-protocols).

### Structural network covariance and individual distance

Regional cortical thickness data from each individual across every ENIGMA cohort was harmonized using neuroComBat (Fortin et al. 2018), a batch-effect correction tool that uses a Bayesian framework to improve the stability of parameter estimates. Harmonization was conducted on mean cortical thickness values with site as the primary nuisance variable (“*batch”* vector), while preserving the effects of diagnosis, age, and sex. Missing data were imputed for individuals with less than 10% missingness using IterativeImputer in the scikit-learn Python toolbox (https://scikit-learn.org/stable/index.html). Before calculating group-level structural covariance networks for schizophrenia and controls, we regressed out the effects of sex and age from the harmonized cortical thickness data. Group-level covariance matrices were then computed separately for schizophrenia and control groups. For individual-level analyses, consistent with previous studies (Sha et al. 2022; Yun et al. 2020), we constructed regional difference matrices by normalizing cortical thickness values relative to the control group mean and standard deviation. The formula is shown in Fig. 2A.

### Organization gradients

Given previous studies have used different sparsity densities ranging from 8% to 40% (Hettwer et al. 2022; Larivière et al. 2022; Sha et al. 2022; Valk et al. 2020), we sparsified the group-level covariance matrices across thresholds from 10% to 40%, in 1% increments, to evaluate group-level template in controls as well as in SCZ. Regarding the case-control comparisons using individual difference matrices, we tested sparsity of 10%, 20% (main analysis in the Results), 30%, and 400%. Organization gradient axes were obtained using diffusion map embedding on the affinity matrix of the sparse covariance matrix using Python toolbox BrainSpace (Vos de Wael et al., 2020). We used alpha = 0.5 to balance the density and geometry of the matrix, which approximates the Laplace–Beltrami operator of the underlying manifold (Coifman et al. 2005).

This approach was described by (Margulies et al. 2016), who used a functional connectivity matrix. When applying this approach to cortical thickness (Valk et al. 2020), a previous study has discovered two important axes: anterior-posterior and inferior-posterior, aligning with dual origins of cortical development. In our study, we can replicate the two axes in controls and schizophrenia. We then applied a sparsity threshold of 0.8 (retaining the top 20% of covariance values) to compute individual cortical gradients, consistent with a prior ENIGMA study (Hettwer et al. 2022). To make individuals comparable, we used Procrustes rotation to align individual gradients to the group-level template generated from control data. The first two gradients (G1 and G2) were then used for subsequent analyses, which accounted for 20.4% and 17.2% of variance in controls, and 21.7% and 17.7% in schizophrenia, respectively.

### Small-world topology

Small-world topology was characterized by two nodal indices derived from the thresholded cortical thickness covariance matrix, nodal clustering coefficient (CC) and shortest path length (SPL), computed for each cortical region. CC is a regional averaged score of edges and SPL is computed for each node as the mean shortest path length to all other nodes in the network. Regional SPL is interpreted as a measure of network integration, complementary to the CC, which reflects local segregation. To make individuals comparable, scores were normalized to 0–1 for each individual. As informed by previous work, three different states were used to characterize topology, including: regular (high CC and long SPL), small-world (balance between CC and SPL), and randomized (low CCand short SPL) (Larivière et al. 2022; Sha et al. 2022).

### Case-control comparisons

In the case-controls comparison, we used linear regression models with *brain ~ age + sex + DX* to obtain the statistical estimates. A false discovery rate (FDR) (Benjamini and Hochberg 1995) correction (*q* < 0.05) was then applied for brain features within each metric (G1, G2, SPL, and CC), separately. Furthermore, ICV was included as a covariate in separate models to test robustness of the group effects (**Supplementary Fig. S4)**. Interactions between diagnosis and age/sex were performed using *brain ~ age + sex + DX + age × DX* and *brain ~ age + sex + DX + sex × DX*. Disease duration effects in schizophrenia were examined using *brain ~ age + sex + duration*.

### Brain-symptoms association

Brain-symptom associations were examined in the subsample of individuals with schizophrenia with available item-level PANSS data (n = 881), representing 22% of the full schizophrenia sample (n=3,958). Partial least squares (PLS) was used to associate clinical symptoms with brain features in schizophrenia (McIntosh and Lobaugh 2004). The significance of each latent variable was assessed by bootstrap resampling testing (*n* = 5,000), with all reported latent variables significant at *P* < 0.001. Bootstrap resampling was used to assess the reliability of individual feature loadings contributing to these significant latent variables. As individual loadings did not survive at the conventional 95% confidence level, we report feature-level contributions at the 90% confidence interval as an exploratory characterization of the dominant contributors to the established brain-symptom association. Clinical symptoms are presented at the factor level in the main figures and at the item level in the supplementary figures. PANSS symptoms were grouped according to the five-factor model reported by (Lim et al. 2021). The following dimensions were identified positive (6 items), negative (8 items), cognitive/disorganization (6 items), depression/anxiety (3 items), and hostility (5 items) using exploratory factor analysis in a large multi-ethnic schizophrenia sample and validated against a meta-analysis of published PANSS models. Two PANSS items were not assigned.

### Subtyping

A *k*-means algorithm was used to cluster SCZ individuals using significant brain features and the SSE was calculated for each different cluster solution (2 through 9). However, no clear convergence was observed in the SSE curve, therefore, principal components analysis (PCA) was applied to reduce the dimensionality of the brain features;the first two PCs explained 9.18% and 6.13% of the total variance, respectively (Fig. 3B). In the space defined by the first two PCs, a two-subtype solution provided the best separation. The sample was then split into two subsamples for cross-validation of the subtyping approach, yielding a high assignment concordance in both subsamples (> 98%).

### Normative models

Generative additive models (GAM) were used to generate the population models and deviation scores using the Python toolbox pyGAM (https://pygam.readthedocs.io/en/latest/).

Models were fit in controls, with age specified as a smooth term, sex as a categorical factor, and ICV as a linear covariate, to estimate mean and regional cortical thickness. For each region, we derived the predicted mean and 95% CI, and computed deviation scores as *(observed - predicted cortical thickness) × 4 / (upper - lower 95% CI)*, approximating a *z*-score.

### Spatial autocorrelation null models

To account for spatial autocorrelation between cortical maps, we used spin permutation tests to obtain corrected *P*-values (Vos de Wael et al. 2020). The DK cortical values were projected onto the fsaverage5 surface and the surface was randomly rotated 1,000 times to generate surrogate maps. Null distributions were constructed from these rotations, and *P*-values were calculated as the proportion of null models exceeding the empirical statistic. Analyses were conducted using the ENIGMA toolbox (Larivière et al. 2021).

## Data Availability

The neuroimaging data used in this study were obtained through the ENIGMA consortium and are available in accordance with ENIGMA data-sharing policies. Source data for the figures and region-specific statistics are provided with this paper.

## Code availability

All the analytic scripts and visualization are openly available at a GitHub repository (https://github.com/wanb-psych/enigma_scz). The python packages are completely open for use, see documentations: BrainSpace (https://brainspace.readthedocs.io/en/latest/) for gradients construction, NetworkX (https://networkx.org/) for small-world topology construction. Scikit-learn (https://scikit-learn.org/stable/) for PLS construction, Statsmodels (https://www.statsmodels.org/stable/index.html) for the statistics, and ENIGMA toolbox (https://enigma-toolbox.readthedocs.io/en/latest/) for spatial autocorrelation null models (spin test).
